# Spatial and temporal dynamics of malaria in Madagascar

**DOI:** 10.1186/s12936-018-2206-8

**Published:** 2018-02-01

**Authors:** Felana A. Ihantamalala, Feno M. J. Rakotoarimanana, Tanjona Ramiadantsoa, Jean Marius Rakotondramanga, Gwenaëlle Pennober, Fanjasoa Rakotomanana, Simon Cauchemez, Charlotte J. E. Metcalf, Vincent Herbreteau, Amy Wesolowski

**Affiliations:** 1 Epidemiology Unit, Institut Pasteur de Madagascar, Antananarivo, Madagascar; 2 UMR 228 ESPACE-DEV (IRD, UM2, UR, UAG), Saint-Pierre, Reunion France; 3 Department of Integrative Biology, University of Wisconsin-Madison, Madison, WI USA; 4 Mathematical Modelling of Infectious Diseases Unit, Institut Pasteur, 75015 Paris, France; 5 Centre National de la Recherche Scientifique, URA3012, 75015 Paris, France; 6 Centre of Bioinformatics, Biostatistics and Integrative Biology, Institut Pasteur, 75015 Paris, France; 7 Department of Ecology and Evolutionary Biology, Princeton University, Princeton, NJ USA; 8 Woodrow Wilson School of Public Affairs, Princeton University, Princeton, NJ USA; 9 Department of Epidemiology, Johns Hopkins Bloomberg School of Public Health, Baltimore, MD USA

**Keywords:** Malaria, Madagascar, Strata, Standardized Incidence Ratio, SaTScan

## Abstract

**Background:**

Malaria is one of the primary health concerns in Madagascar. Based on the duration and intensity of transmission, Madagascar is divided into five epidemiological strata that range from low to mesoendemic transmission. In this study, the spatial and temporal dynamics of malaria within each epidemiological zone were studied.

**Methods:**

The number of reported cases of uncomplicated malaria from 112 health districts between 2010 and 2014 were compiled and analysed. First, a Standardized Incidence Ratio was calculated to detect districts with anomalous incidence compared to the stratum-level incidence. Building on this, spatial and temporal malaria clusters were identified throughout the country and their variability across zones and over time was analysed.

**Results:**

The incidence of malaria increased from 2010 to 2014 within each stratum. A basic analysis showed that districts with more than 50 cases per 1000 inhabitants are mainly located in two strata: East and West. Lower incidence values were found in the Highlands and Fringe zones. The standardization method revealed that the number of districts with a higher than expected numbers of cases increased through time and expanded into the Highlands and Fringe zones. The cluster analysis showed that for the endemic coastal region, clusters of districts migrated southward and the incidence of malaria was the highest between January and July with some variation within strata.

**Conclusion:**

This study identified critical districts with low incidence that shifted to high incidence and district that were consistent clusters across each year. The current study provided a detailed description of changes in malaria epidemiology and can aid the national malaria programme to reduce and prevent the expansion of the disease by targeting the appropriate areas.

**Electronic supplementary material:**

The online version of this article (10.1186/s12936-018-2206-8) contains supplementary material, which is available to authorized users.

## Background

Malaria remains a key global health concern and leading cause of mortality and morbidity worldwide. Global control efforts have resulted in a large reduction in the morbidity of the disease down from 13% in 2005 to 9% in 2015 [1]. Despite this progress, there are still an estimated 214 million cases and 438,000 deaths in 2015, primarily in sub-Saharan Africa [2]. Within Madagascar, malaria remains a serious public health issue and a leading cause for seeking-care at health facilities, although incidence initially declined at the beginning of the century (see Additional file 1) [3–5]. Although four different species of malaria in humans have been observed within the country, *Plasmodium falciparum* remains the most common cause of illness in children [4].

Malaria control throughout the country is organized through and performed by the National Malaria Control Programme (NMCP). The control program has focused on indoor residual spraying (IRS), distribution of insecticide-treated nets (LLITNs), intermittent preventative treatment for pregnant women (IPTp) and overall improved access to diagnostics and drug treatments [6–8] with the majority of these interventions available since 2008. Coverage of these interventions varies across the country, for example IPTP is primarily provided in endemic areas. LLINs are available throughout the country with three large distribution campaigns performed between 2009 and 2015 with coverage estimated at 82% in endemic areas and 38% in low transmission areas [9]. IRS has focused on areas with lower transmission (Highlands and Fringe) with spraying done in five different regions. Routine coverage, of which malaria diagnostic and treatment is a key component, is provided through a national system comprising of hospitals, local health clinics and community health workers. Since 2007, the country has relied on a network of community health workers in 375 communes across the country to assist in the management of uncomplicated malaria [10–12].”

Since 2009, the number of cases and local epidemics have continued to increase and this has resulted in changes in the spatial patterns of malaria [13, 14]. The political and economic crisis in 2009 resulted in a lack of funds and interruption in supplies to health facilities which caused health facilities to scale back the malaria control programmes efforts [6]. In conjunction with economic changes, natural factors including climate change [15], mosquito vector behaviour and resistance to insecticides [16], and a weakening of natural immunity [17–19] are also possible causes for the change in incidence. This increase in the number of malaria reports continues to present day (see Additional file 1) [20].

**Fig. 1:**
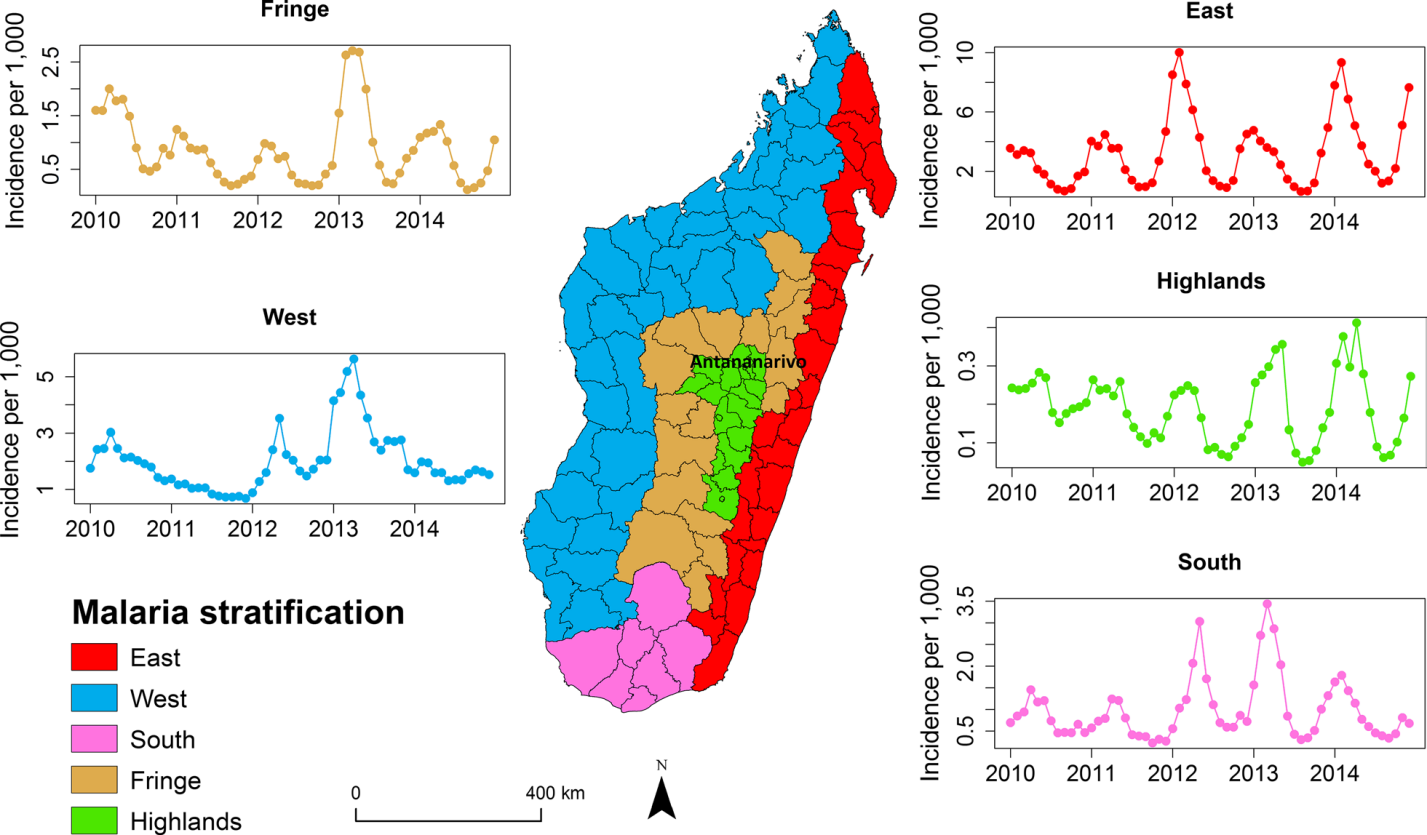
The malaria stratification zones and reported incidence in Madagascar. The health districts (outlined in black) report the number of confirmed (by RDT) cases of malaria per month (Additional file 2). In each of the five zones (West—blue, East—red, Highlands—green, South—purple, and Fringe—orange), the time series of reported monthly incidence from 2010 to 2014 per strata is shown on this figure

Although the national trends have been documented, in order to optimally target control efforts, an understanding of the local heterogeneity in incidence is necessary [21]. Currently, malaria control efforts are stratified according to five geographically continuous zones (East, West, South, Highlands, and Fringe) roughly based on the magnitude of malaria transmission (see Fig. 1). The coasts are divided into two endemic zones that vary in their transmission season: East (perennial transmission) and West (seasonal transmission). The South is characterized by a dry and hot climate prone to episodic outbreaks with little sustained transmission. The Central Highlands are geographically divided into two areas both with low, unstable transmission: however, these zones represent broad geographic and population areas and an effective control strategy will still require an understanding of the spatiotemporal distribution of the disease within these strata. For example, if locations within a stratum have highly heterogeneous malaria epidemiological characteristics, a single control policy for this area based on the mean transmission may underserve high transmission areas. Moreover, if key transmission hotspot areas can be identified, these locations could be targeted to reduce transmission intensity that may be impacting other low transmission districts through the importation of parasites by human travel [22].

In this study the dynamics of malaria in Madagascar between 2010 and 2014 were analysed to derive a spatially refined characterization of malaria spatial distribution in Madagascar with a view to inform control strategies. Building off recent work by Howes et al. this study analyses additional heterogeneities of malaria transmission within Madagascar [13]. Using reported cases of uncomplicated malaria from the Health Management Information System (HMIS), first the incidence of malaria nationally was analysed and then a Standardized Incidence Ratio (SIR) was used to identify districts with a higher than expected number of cases based on their stratification zone. Next, a clustering method was used to identify high transmission clusters both spatially and temporally. In particular, how the current strata definitions may or may not accurately reflect the actual spatio-temporal dynamics of malaria throughout the country was investigated. This approach makes it possible to subdivide the large spatially homogenous malaria control zones into more targeted regions using the spatial and clustering of incidence.

## Methods

### Data

The HMIS provided information on uncomplicated malaria from 2010 to 2014 across the country (see Additional file 2). These data are recorded monthly by the HMIS from each primary and secondary health facility and aggregated to the health district (N = 112) [23]. This study analysed the aggregated district-level data. Only the completeness rates of all reports per health centres were available in 2010 and 2014 and were respectively 93% [24] and 89% [3] suggesting that the majority of health centres report regularly. Malaria cases are grouped into five age classes: less than 1-year-old, between 1 and 4-year-old, between 5 and 14-year-old, between 15 and 25-year-old, and above 25-year-old. To further put these results in context with current malaria case reports, the number of nationally reported cases in 2015 and 2016 from the World Malaria Report in 2017 was also used [20]. The National Institute of Statistic (INSTAT) further provided population and demographic data from 2010 to 2014 per district. Since the last population census in Madagascar dates from 1993, the INSTAT estimates yearly population with a fixed 2.8% national annual growth rate. These data are further grouped by 5-year age classes [25].

This study builds on recent work on this question by Howes et al. who used modelled prevalence maps of *P. falciparum* to identify transmission regions (by contrast with the NMCP strata) by directly investigating how the spatial, temporal, and spatio-temporal dynamics independently would identify clusters of districts with similar epidemiological patterns [13]. This study further focused on how either spatial or temporal dimensions of incidence identify different clusters of districts with similar epidemiological characteristics. Unlike previous work, this study also investigated how these clusters have changed over time highlighting the changing nature of malaria epidemiology that may help refine the target area for malaria control and potential outbreaks.

### Crude incidence analysis

First, the national annual incidence of malaria for each health district were analysed. For mapping purpose, the incidence per 1000 individuals was grouped into four categories that correspond to the NMCP elimination phase classifications: less than 1 (pre-elimination), 1 to less than 10 (moderate transmission), 10 to less than 50 and 50 and above (high transmission) [7].

### Standardized incidence analysis

Considering that young people are the most affected by malaria in endemic areas and that the age-structure of the population varies across the country, the standardized incidence was calculated by taking into account age structure. The indirect Standardized Incidence Ratio (SIR) method was used which is based on the population size and the distribution in each age class per district [26–28]. For this analysis, both malaria and population data were grouped into four overlapping age classes: 0–4, 5–14, 15–25 years old, and above 25 years old. Stratification-level incidence was calculated for each district *i* and each age class *j* using: $\hat{p}(j) = \sum\nolimits_{i} {D_{i} \left( j \right)} /\sum\nolimits_{i} {N_{i} \left( j \right)}$ where *D*_*i*_(*j*) and *N*_*i*_(*j*) are the number of malaria cases and population size for district *i* for age class *j,* respectively. The expected number of cases for age class *j* in a district *i* is $\hat{D}_{i} (j) = \hat{p}\left( j \right)N_{i} \left( j \right)$, and the total number of expected cases is $\hat{D}_{i} = \sum\nolimits_{j = 1}^{4} {\hat{D}_{i} (j)}$. Finally, the SIR index for a district *i* is obtained by the following equation:

$${\text{SIR}}_{i} = \frac{{D_{i} }}{{\hat{D}_{i} }}$$

Districts with anomalous SIR values relative to the other districts in the same stratification zone were identified. Intuitively, a SIR larger (or smaller) than 1 means that the observed cases are higher (or lower) than what would be expected given its population size and structure. The numerical value of SIR allows us to quantify the magnitude of the difference. The confidence interval was calculated for each district assuming that SIR is a composite of Poisson and Chi squared distribution [29–31], and thus approximated the lower (LSIR) and upper (USIR) limit of the 95% confidence interval of that distribution by

$${\text{LSIR}}_{i} = \frac{{D_{i} }}{{\hat{D}_{i} }}\left( {1 - \frac{1}{{9D_{i} }} - \frac{1.96}{{3\sqrt {D_{i} } }}} \right)^{3}$$

and

$${\text{USIR}}_{i} = \frac{{D_{i} + 1}}{{\hat{D}_{i} }}\left( {1 - \frac{1}{{9\left( {D_{i} + 1} \right)}} + \frac{1.96}{{3\sqrt {D_{i} + 1} }}} \right)^{3} .$$

SIR_*i*_ is significantly larger or smaller than 1 if SIR_*i*_ > USIR_*i*_ or SIR_*i*_ < LSIR_*i*_. For ease of interpretation, SIR values were classified into six categories. A district has a *high*-risk, *higher*-risk, or *highest*-risk if the number of cases is one to less than two times higher (1 < SIR < 2), two times to less than four times higher (2 ≤ SIR < 4), or four times higher (4 ≤ SIR) than the number of expected cases, respectively. Conversely, a district has a *low*-risk, *lower*-risk, or *lowest*-risk if the number of observed cases is half to less than one times lower (0.5 ≤ SIR < 1), quarter to less than half times if (0.25 ≤ SIR < 0.5), or less than a quarter times (SIR < 0.25) the number of expected cases, respectively.

### Cluster analyses

Using the previous SIR analyses, clusters of districts that had a higher than expected incidence of malaria were identified using the magnitude of cases. For each stratum and year, clusters in both space, time, and space–time were identified using SaTScan version 9.4.4 [32, 33].

#### Spatial cluster analysis

A cluster is identified if the observed incidence in a given area exceeds the number of expected cases [34, 35]. For each district centroid, increasing radii are chosen that form a circular window centered at the district centroid. The minimum radius is set to zero—this would only include the district centroid. The maximum radius is chosen to include at most 50% of the district population at risk within each stratum. Based on the circular window, all districts that intersect the window are included in a cluster. A cluster is defined if the observed number of cases exceeds the expected number. The expected number of cases used as a benchmark to define a cluster assumes that the incidence is the same for all districts with the stratum. The expected versus observed number of cases is compared using a log-likelihood ratio test assuming a Poisson distribution. The alternative hypothesis is that the risk is higher inside than outside the window. The primary cluster is the district or grouping of districts with the highest log-likelihood ratio, calculated using a Monte Carlo simulation.

#### Temporal cluster analysis

Next, temporal clusters were identified by which months districts have similarly higher than expected incidence values. This approach is similar to the spatial analysis, but instead uses time (within the same time window) centered at each month to identify clusters. A cluster is identified if the observed incidence in a given set of months exceeds the number of expected cases within that window of time (with a maximum window size of six months).

#### Space–time cluster analysis

Space–time clustering consists of scanning across space and time using a cylindrical window where the base is centered at the centroid of each district and the height corresponds to time aggregation of 1 month. The principle is similar to spatial clustering with at most 50% of the total population allowed in a single cluster. The space–time clusters were detected if the number of observed cases with a time window (range 1–6 months) exceeded significantly that of the expected based on the values within the stratification zone.

## Results

### Crude incidence analysis

The national analysis confirmed that the crude incidence of malaria in Madagascar increased between 2010 and 2016 from 14 per 1000 to 20 per 1000. The highest incidence was in 2015 with a value of 32 per 1000 and the lowest was 12 per 1000 in 2011 (Additional file 1). The incidence is also spatially heterogeneous with different seasonal patterns across the country (Fig. 1). Overall, the highest incidence values were in the East, which also receives the largest amount of rainfall throughout the country [36], with minimum of 0.64 per 1000 in August 2013 and the maximum 10 per 1000 in February 2012, followed by the West between 0.67 per 1000 in December 2011 and 5.63 per 1000 in April 2013, and the South with minimum of 0.23 per 1000 in October 2011 to 3.44 per 1000 in March 2013. The Fringe varied between 0.12 per 1000 in August 2012 to 2.71 per 1000 in March 2013 and has intermediate incidence values. In the Highlands that consistently has the lowest values, the minimum value of incidence is 0.05 per 1000 in August 2013 and the maximum is 0.41 per 1000 in April 2014. Incidence in the East, Highlands, South, and Fringe peaked between January and May, with the lowest values from July to September. For the West and Fringe, the peak of incidence was in 2013 in May and in April respectively—April 2012 and March 2014 in the East—June 2013 and May 2014 in the Highlands and in May 2012 and April 2013 in the South. The incidence per age-class is the highest in children under 15 years old in all areas aside from the Central Highlands (see Additional file 2). In these areas, the temporal trends in age-specific incidence was consistent with the broader regional pattern. However, in the Highlands there was increased temporal variability amongst age classes.

**Fig. 2:**
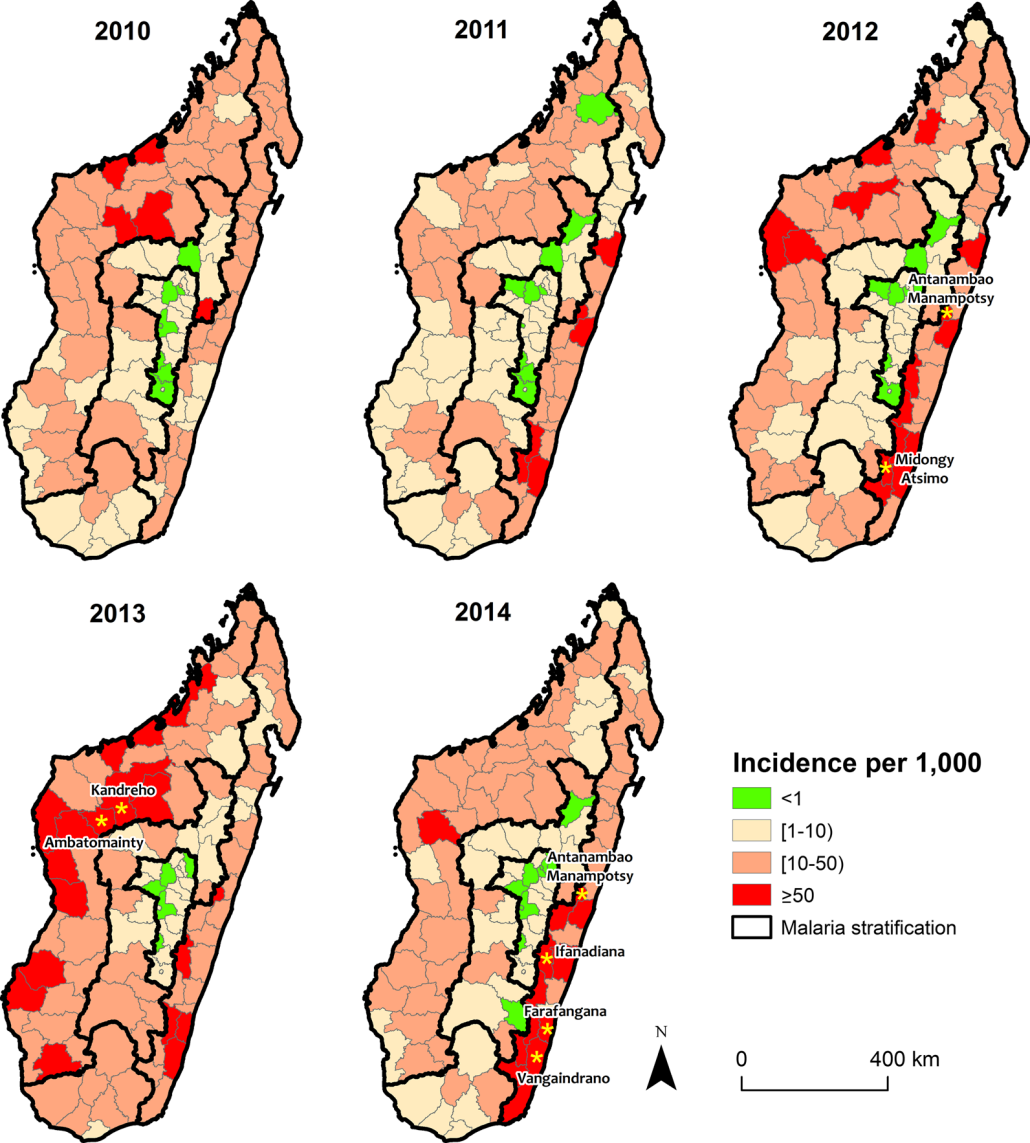
The annual incidence per 1000 of malaria from 2010 to 2014 per district. Malaria incidence was classified into four groups according to the malaria elimination phase: pre-elimination—green, moderate and high transmission—shades of red. There is substantial heterogeneity in incidence per strata (outlined in black) regarding crude incidences over the years. Yellow stars represent district with incidence above 100 per 1000. Consistently, the lowest incidence of malaria was found in the Fringe and the Highlands. Most of district colored in red are in the coastal zone (East and West)

The incidence per year in the majority of districts (~ 53/112 districts) ranged from 10 to 50 per 1000. The highest incidence districts (≥ 50 per 1000) were found along the coasts (West and East), and the magnitude of incidence remained fairly consistent between years (Fig. 2). From 2010 to 2014, the number of high incidence (≥ 50 per 1000) districts increased in the West, whereas it remained stable in the East, although the location of high incidence districts varied. Consistently, the Highlands stratum that includes the capital city of Antananarivo (Fig. 1) had the lowest incidence districts with many of the districts (7/20 in 2010 and 10/20 in 2014) considered in the pre-elimination phase.

### Standardized incidence analysis

**Fig. 3:**
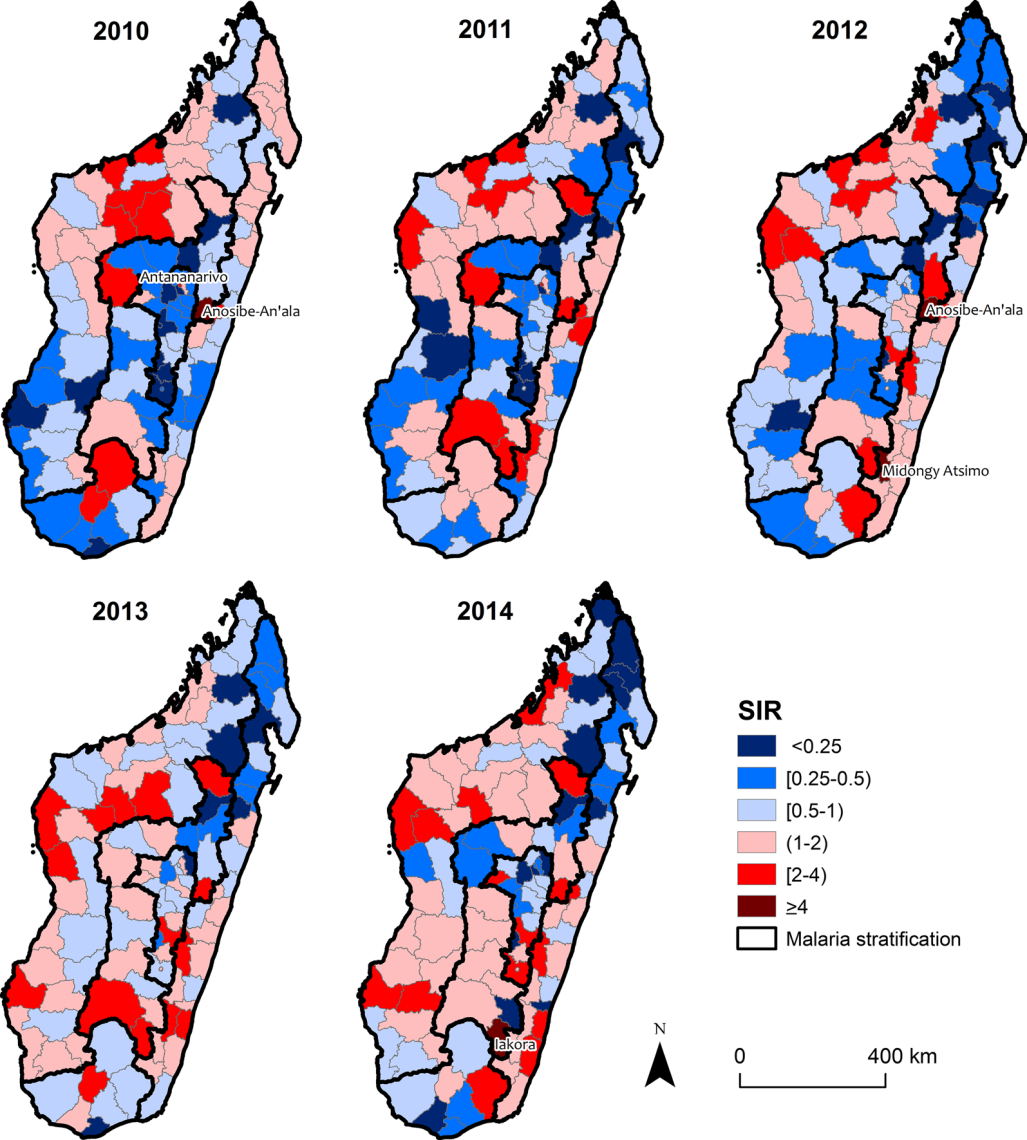
The Standardized Incidence Ratio per year. These figures show the intensity’s degree of malaria incidence per district. The value of SIR is the result of the ratio between the number of observed cases and the expected cases on each stratum. District with high and low incidences relative to the background of whole strata are shown in shades of red and blue, respectively. Malaria strata are delimited by the thick black line. Some districts in low incidence (SIR < 1) shifted to high incidence in the Highlands and Fringe. In the East and West, the high incidence was in the north of each stratum in 2010 and shifted south in 2014

The Standardized Incidence Ratio (SIR) was used to identify how malaria patterns changed within each stratum since 2010. Overall, the number of high-risk districts increased from 41 in 2010 to 53 in 2014 (Additional file 3). Within each stratum, the location of high-risk districts are spatially heterogeneous with an overall concentration in the southern area of each stratum (Fig. 3). In the South and Fringe, there was no clear spatial aggregation of high or higher than expected risk districts (Fig. 3). In the Highlands, there were consistently many *low*-risk districts and few *high*-risk districts. Only the capital district of Antananarivo was classified as *higher*-risk in 2010 (SIR = 3.82; 95% CI 3.76–3.87) and 2011 (SIR = 3.12; 95% CI 3.07–3.19), three new districts joined that category by 2014. In the West, the number of districts that were *high*-risk increased from 15 in 2010 (located in the centre) to 23 in 2014 (located in the south) suggesting that behind the overall decrease in the risk at national level there is a dispersion of cases through the strata (Additional file 3). In the East, the number of *highest*-*risk* districts also increased where the north–south gradient of the distribution from *high*-risk to *low*-risk districts in 2010 flipped and by 2014 the majority of *high*-risk districts occur in the southern part of the East stratification (see Additional file 3).

### Spatial clustering

**Fig. 4:**
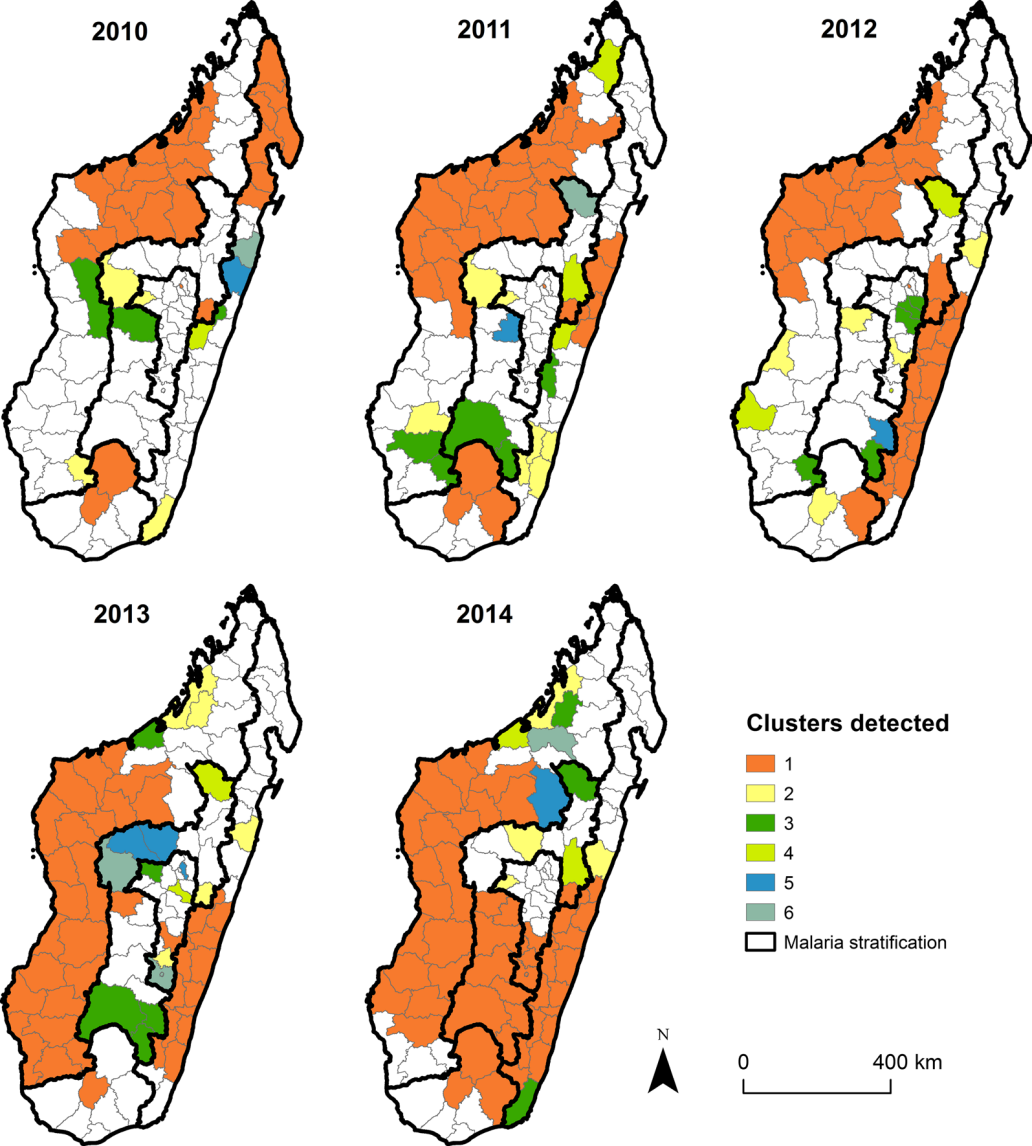
The spatial clustering of malaria per year. The primary clusters (1) are shows in orange, the secondary clusters (2) are show in yellow, the third cluster (3) and the fourth (4) are in shades of green, the fifth (5) and sixth are in shades of blue (6). Clusters was done on SIR ratios and the cluster ranks is based on the value of log-likelihood ratio with p value < 0.05, the one or the group of district which have the highest value is consider the primary cluster

Next, spatial clusters of districts with higher than expected numbers of cases were identified (Fig. 4). In each stratum per year, we detected between one and six spatial clusters which represent 10% to 71% of the districts within a stratum across years and across strata (Fig. 4). A primary cluster was designated as the district or the groups of districts (shown in orange) with the highest likelihood raito value with *p* value < 0.05 in each stratum per year, and as secondary clusters the groups of non-contiguous districts that also had a significantly higher log-likelihood ratio after the primary cluster. From 2010 to 2014, the number of districts included in the primary cluster increased as a result of increasing patterns of risk.

The largest changes in the West and East strata were identified. In both strata, the primary cluster increased in size and shifted in location. Specifically, the primary cluster has shifted from the northern to the middle-southern districts. The number of districts included in the primary cluster increased in the East (from 8 districts in 2010 to 15 districts in 2014) and in the West (from 15 districts in 2010 to 19 districts in 2014). The number of districts included in the secondary clusters were usually inconsistent varying from two to five and often consisted of one or two districts. In the South, few clusters were identified that only included a small number of districts (see Additional file 4) suggesting that incidence within this stratum is more spatially homogenous. Spatial clusters in the Fringe and Highlands were scattered across the stratum between 2010 and 2014 with a primary cluster observed in each year. One key exception in the Fringe was a cluster made of a single district (Anosibe-an’Ala) that was identified each year. That district had a higher incidence relative to the other districts in the Fringe, but it neighbours a higher incidence district (Antanambao Manampotsy) located in the East stratum suggesting that the stratum borders may not accurately reflect incidence patterns.

### Temporal clustering

Next, the temporal clustering of districts per year were analysed to identify periods with a higher than expected reported number of cases. For each stratum, a temporal cluster was identified if there was the same seasonal pattern of high malaria incidence between districts in a given year. In the East, the temporal cluster decreased in length (January–June in 2010 to January–March in 2014) (Table 1). In the West, the season between years was more erratic with clusters identified between January and July, although this varied by year. For example, the temporal cluster in 2011 had a long temporal range from January to June, however by the next year the peak season was much shorter (April–June) that only encompassed half the number of months. Overall, the temporal cluster in the South has shifted earlier in the year from February–June in 2010 to January–April in 2014. In both the Fringe and Highlands stratum, the cluster remained consistent among the years and includes the longest season (January–June) with small spatial and temporal variability although it was expected.

**Table 1:** Malaria clustering using the retrospective temporal analysis

Stratum	Year	Time frame	Observed cases	Expected cases	RR	LLR	p value
East	2010	January–June	80,029	57,009.05	2.33	9460.98	0.001
	2011	January–May	89,233	65,968.38	1.80	6860.41	0.001
	2012	January–April	152,613	83,303.54	3.11	39,813.28	0.001
	2013	January–April	84,064	57,507.62	1.89	86,18.87	0.001
	2014	January–March	128,165	75,309.53	2.21	21,888.52	0.001
West	2010	February–July	62,405	5,0907.61	1.58	2594.22	0.001
	2011	January–June	30,105	2,4281.27	1.62	1396.91	0.001
	2012	April–June	36,545	2,4390.73	1.79	3684.61	0.001
	2013	February–June	97,494	73,479.45	1.72	6528.77	0.001
	2014	February–March	15,949	12,562.15	1.34	509.90	0.001
South	2010	February–June	6970	4870.27	2.05	752.07	0.001
	2011	February–June	6097	3883.56	2.61	1050.38	0.001
	2012	April–June	9747	5023.40	2.82	2563.01	0.001
	2013	January–May	17,980	10,078.53	3.99	5266.30	0.001
	2014	January–April	8746	5041.32	2.71	1878.97	0.001
Fringe	2010	January–June	24,482	17,763.29	2.19	2577.61	0.001
	2011	January-June	14,134	9089.67	3.42	2926.86	0.001
	2012	January–May	9400	6168.46	2.43	1422.14	0.001
	2013	January–May	27,258	15,265.07	4.01	8011.42	0.001
	2014	January–May	13,222	7770.09	3.37	3229.23	0.001
Highlands	2010	January–June	16,556	13,839.81	1.48	531.55	0.001
	2011	January–June	12,161	9779.45	1.64	580.18	0.001
	2012	January–May	7504	4978.21	2.36	1076.06	0.001
	2013	January–May	9092	5479.44	3.10	2004.89	0.001
	2014	January–May	10,199	6629.93	2.48	1608.58	0.001

*RR* relative risk, *LLR* log likelihood ratio

### Space–time clustering

**Fig. 5:**
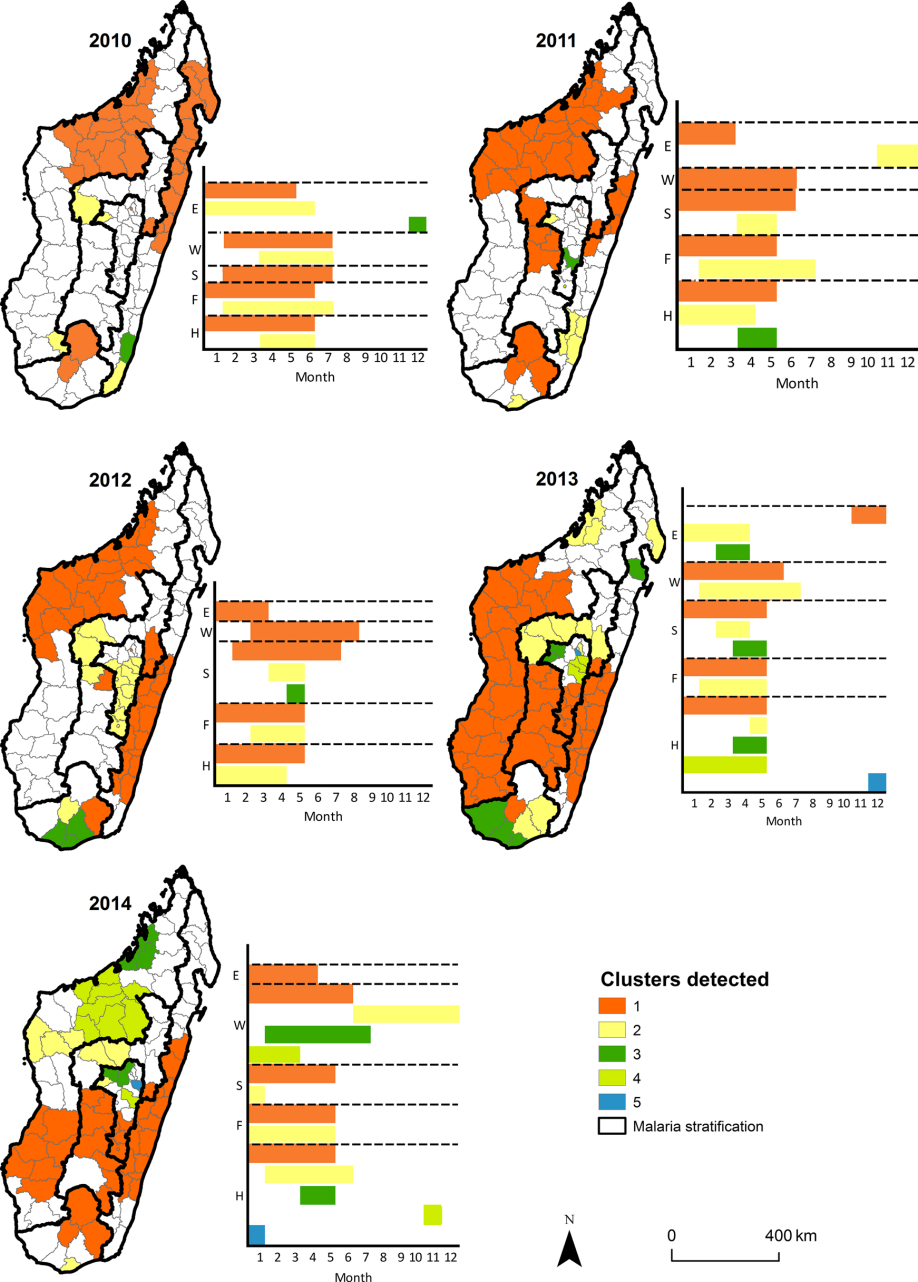
The space–time clustering of malaria per year. The primary clusters (1) are shown in orange, secondary clusters: (2) in yellow, (3) in green, (4) and (5) in shades of blue, as in Fig. 4. This figure represents the time of occurrence per month and the spatial distribution of each cluster per year

Finally, both spatial and temporal clusters of high incidence districts per zone were identified (Fig. 5, Additional file 5). In the majority of strata, the timing of the space–time clusters occurs during the same period of the year suggesting that although the spatial location of the cluster may vary, the season is fairly consistent within the stratum. The notable exception is the West stratum that shows distinct spatial and temporal differences between the identified clusters (Fig. 5). For example in 2014, there is no temporal overlap between the primary (located in the southern part of the stratum) and secondary cluster (located in the centre of the stratum).

In the South, the number of districts composing the space–time clusters, from 2010 to 2013 between January and July, increased from two to six. In 2014, only four districts were left: the primary cluster composed of three districts between January and May, and a secondary cluster composed of one district in January. For the Fringe, only two clusters were detected each year. The largest number of clusters was found in 2013: between January and May with nine districts within the primary cluster, and between February and May with five districts within the secondary cluster. The primary clusters generally lasted between January and May. In 2013 and 2014, the Highlands had the largest number of space–time clusters compared to the other stratifications with one primary cluster spread out between January and May composed of six and five districts. Four secondary clusters were detected with different appearance through the year for these 2 years.

In the East, the appearance of primary clusters changed considerably with time: between January and May in 2010 and 2011, between January and April in 2012 and 2014, and between November and December in 2013. The number of districts included in those primary clusters varied between 6 and 15 whereas the number of districts in the secondary clusters varied between zero (no secondary cluster) and six. Evidence of space–time clustering is shown by the excess of observed over expected cases per spatial unit and time-period. As shown in Fig. 5, the study has not only identified the high incidence geographic unit but has also defined their respective period of occurrence. Space–time cluster locations with more recent data available from the World Malaria Report were compared, and found similar smaller clusters observed in 2014, and had higher and similar incidence values in 2016 suggesting that these results could be useful in understanding the current epidemiology of malaria. Using the combined analysis, these results underline that there is both spatial and timing heterogeneity for a given stratum. These results show that in both endemic and low endemic transmission strata, the number of districts at high risk increased between 2010–2014 and occurred between January and July.

## Discussion

Here, the spatio-temporal variability in malaria incidence across epidemiological strata defined by the NMCP were investigated. Districts within Madagascar are separated into coarse malaria epidemiological stratified zones that inform key decisions on malaria control and policy within the country. However, the level of heterogeneity within these zones is unclear, as is the degree to which the constituent districts are spatially and temporally clustered.

First, the spatial distribution of crude incidence of malaria for each district from 2010 to 2014 was mapped. Here, zone wide incidence values reflect the broad endemicity and elimination categories used by the NMCP. The differences in incidence among districts within each stratum were further characterized. An age Standardized Incidence Ratio was calculated for each district per year [37]. This simple measure was used to compare districts within a single stratum, minimizing the bias associated with differential case reporting and diagnostics. Finally, clusters of districts within each stratum using spatial, temporal, and space–time analyses were detected. This study was only able to describe heterogeneity at the district level, not at a finer spatial resolution. Although an additional level of heterogeneity may exist at finer spatial scales, the health centre does not necessarily serve a specific population within these smaller geographic units and age-stratified population data are unavailable at finer scales [25]. Although these data may not be complete for every health facility within every district, they can nonetheless be considered representative since around 90% of health facilities reported consistently from 2010 to 2014 [13].

High risk areas across the country within a given strata over a specified time range were identified using the Kulldorff scan statistic [33]. Clusters are collections of districts with a higher than expected number of cases. This understanding was further extended to include time where temporal clusters imply the mean incidence over the specific time frame is higher than expected. The stratified incidence ratio (SIR) is then able to explore the heterogeneity in incidence between comparable, either geographically or temporal, units [37, 38]. Identifying clusters may assist the NCMP since: (a) high risk areas may be sources of increased clinical burden and (b) possible sources for imported infections in other lower risk areas of the country. Moreover, in low risk districts, the NMCP can utilize these locations to understand the effectiveness of continued control interventions in surrounding areas.

Overall, there is high spatial heterogeneity in incidence between districts within the same stratification zone. This suggests that a more nuanced approach to clustering districts for operational purposes has the potential to significantly strengthen investments in control. In addition, noncontiguous primary spatial clusters in 2010 all became contiguous in 2014, and the number of encompassed districts rose through years. Climate change, particularly warming temperatures, may have played a role in the shift of the distribution of malaria, although additional analyses utilizing data over a longer time frame is necessary to test this hypothesis [15, 39]. Temporal aspects of incidence also could contribute in grouping districts together for malaria control. In the East, Highlands and Fringe, temporal cluster began in January corresponding to the rainy season (Additional file 6).

This study shows that in the East and West strata, zones of high incidence of malaria were aggregated in the north in 2010, but by 2014 high incidence districts were firmly concentrated in the southern portion of these strata. The previously high incidence areas have since become low incidence areas. This change may be partially attributable to the employment of community health workers (CHW) to recognize and manage uncomplicated malaria in children under five in this part of the country [11, 12] although the evidence remains anecdotal.

In contrast, the opposite spatial pattern is observed in the South: previously low incidence areas have since become new high incidence areas, possibly due to decreased usage of insecticide-treated mosquito nets in parts of this zone [14]. The spatial cluster analyses show that in the East and the West, primary clusters consisted of a large block of several districts for each year. This indicates that even if these two strata are characterized by endemic transmission, there are districts within the strata where the number of observed cases is higher than expected.

In the Highlands and Fringe, a few districts that were at a pre-elimination stage worsened into moderate transmission, and the number of *high*-risk districts for these two strata increased. In the capital district Antananarivo, included in the Highlands, the highest reported incidence may result from the introduction of malaria cases from elsewhere in the region given the strong connectivity of this location to other areas of the country [40], whereas low mosquito survival due to unfavourable climate may lead to a limited local transmission [41]. Given the history of incidence in these areas, most individuals may have escaped previous exposure to malaria, and thus have a high susceptibility of being symptomatic when infected [42]. In the Highlands, the highest proportion of cases were in adults 25 years and suggesting evidence of non-locally acquired cases. Further, in these two strata, the space–time clustering method and purely spatial clustering method identified different clusters. The space–time method identified more clusters that were concentrated in the southern part of the region between January and July.

These analyses will be biased by the ability of the routine HMIS data to accurately describe true malaria dynamics within the country. These routinely collected data are subject to reporting biases and heterogeneous healthcare access and coverage. In particular, the biased reporting nature and ability for the HMIS to collect high quality data is a common issue for routinely collected data, particularly those from low income countries [43]. This study focuses on the data from 2010 to 2014 to help mitigated some of these effects. Before 2008, RDTs were not available in the country and diagnosis was done using microscopy, which was not available at all facilities. Instead, the study focused on data post the arrival of RDTs. These data are likely more reliable since the quality of malaria diagnosis was improved. However, temporal and spatial differences in diagnostics still exist and as a result the study focused on comparing incidence within years and within strata as opposed to between years or strata to help reduce some of the biases within these different units.

Similar to a recently published work by Howes et al. [13], substantial heterogeneity within the NMCP stratification zones was identified suggesting that a more nuanced approach to defining district groupings for malaria control may be necessary. This study built upon this work by using a simple age-adjusted incidence rate and spatial–temporal clustering methods to identify how the overall malaria epidemiology has changed from 2010 to 2014 and the overall impact this may have on the understanding of transmission hotspots. The spatial expansion of clusters throughout the country was identified and the geographic shift in high incidence areas towards the southern areas of the country. Retrospectively, clusters in each stratum were identified in 2014. Recent data for 2015 and 2016 seem to reflect the increase in the number of cases at national level. This study highlighted the importance of identifying clusters to help the national programme to better guide these strategic choices. The “increase” in the number of cases within the last 2 years could indeed be related to the expansion and the increased heterogeneity in distribution of malaria in Madagascar.

This retrospective analysis shows that although consistent patterns of transmission are found within each zone, these overall trends likely reflect a smaller number of districts that could be targeted with additional interventions. This work highlights the utility of routinely collected data and insight to be gained using fairly simple clustering analyses. In particular, the detection of malaria clusters in areas where malaria transmission is designated to be in the pre-elimination phase suggests that overall control measures should be reevaluated and strengthened. This analysis also highlights the success of intervention efforts, as the decreasing trend of malaria in the northern part of the country is likely partially attributable to increasing NMCP and partner control efforts.

## Additional files

**Additional file 1.** Incidence per year from 2000 to 2016.

**Additional file 2.** Malaria incidence per age-classes per stratum.

**Additional file 3.** Number of districts per malaria intensity within stratification. The West has the most number of district within stratification and the South the lowest. The number of district with high incidence increased across the year mainly in 2013 within stratification.

**Additional file 4.** Malaria clustering using the retrospective spatial analysis.

**Additional file 5.** Malaria clustering using the retrospective space-time analysis.

**Additional file 6.** Malaria incidence, rainfall and temperature between 2010 and 2014.

## Abbreviations

CHW: community health worker
HMIS: Health Management Information System
INSTAT: National Institute Of Statistic
IPTp: intermittent preventive treatment for pregnant women
IRS: indoor residual spraying
ITN: insecticide-treated nets
LLIN: long-lasting insecticidal net
LSIR: Low Limit of Standardized Incidence Ratio
NMCP: Madagascar national malaria control programme
SIR: Standardized Incidence Ratio
USIR: Upper Limit of Standardized Incidence Ratio
