# Supplementary figures and images for: Spatial and temporal dynamics of malaria in Madagascar
Source: Malar J. 2018 Feb 1;17:58. doi: 10.1186/s12936-018-2206-8 (PMC5796477; doi:10.1186/s12936-018-2206-8)

### Additional file 1: Incidence per year from 2000 to 2016

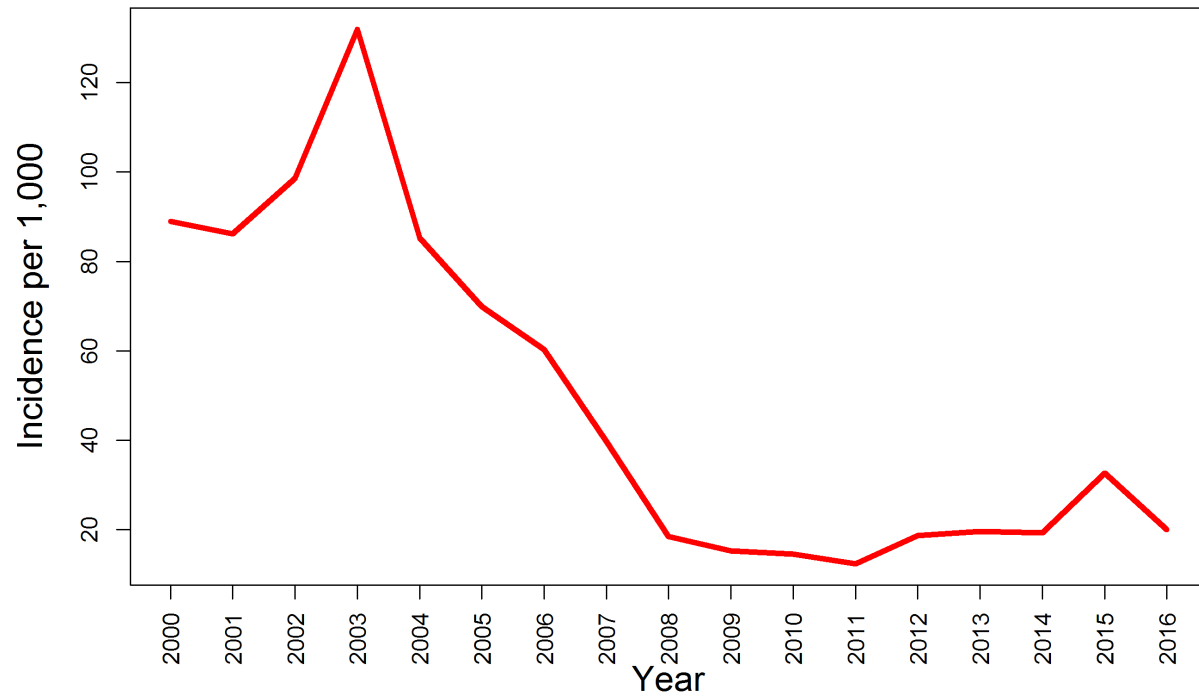

Supplement: Supplementary file 1 — Additional file 1. Incidence per year from 2000 to 2016. [file 12936_2018_2206_MOESM1_ESM.pdf]

## Additional file 2: Malaria incidence per age-classes per stratum

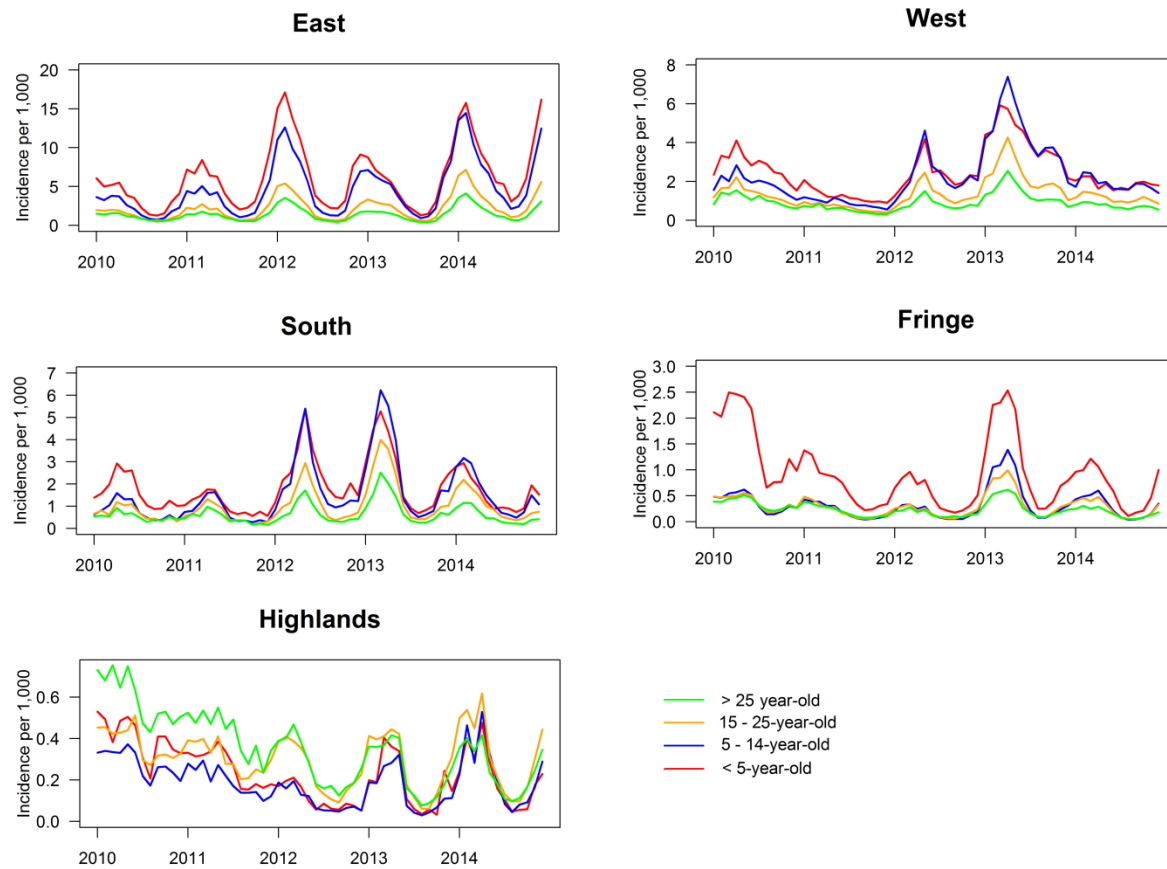

Supplement: Supplementary file 2 — Additional file 2. Malaria incidence per age-classes per stratum. [file 12936_2018_2206_MOESM2_ESM.pdf]

## Additional file 6: Malaria incidence, rainfall and temperature between 2010 and 2014

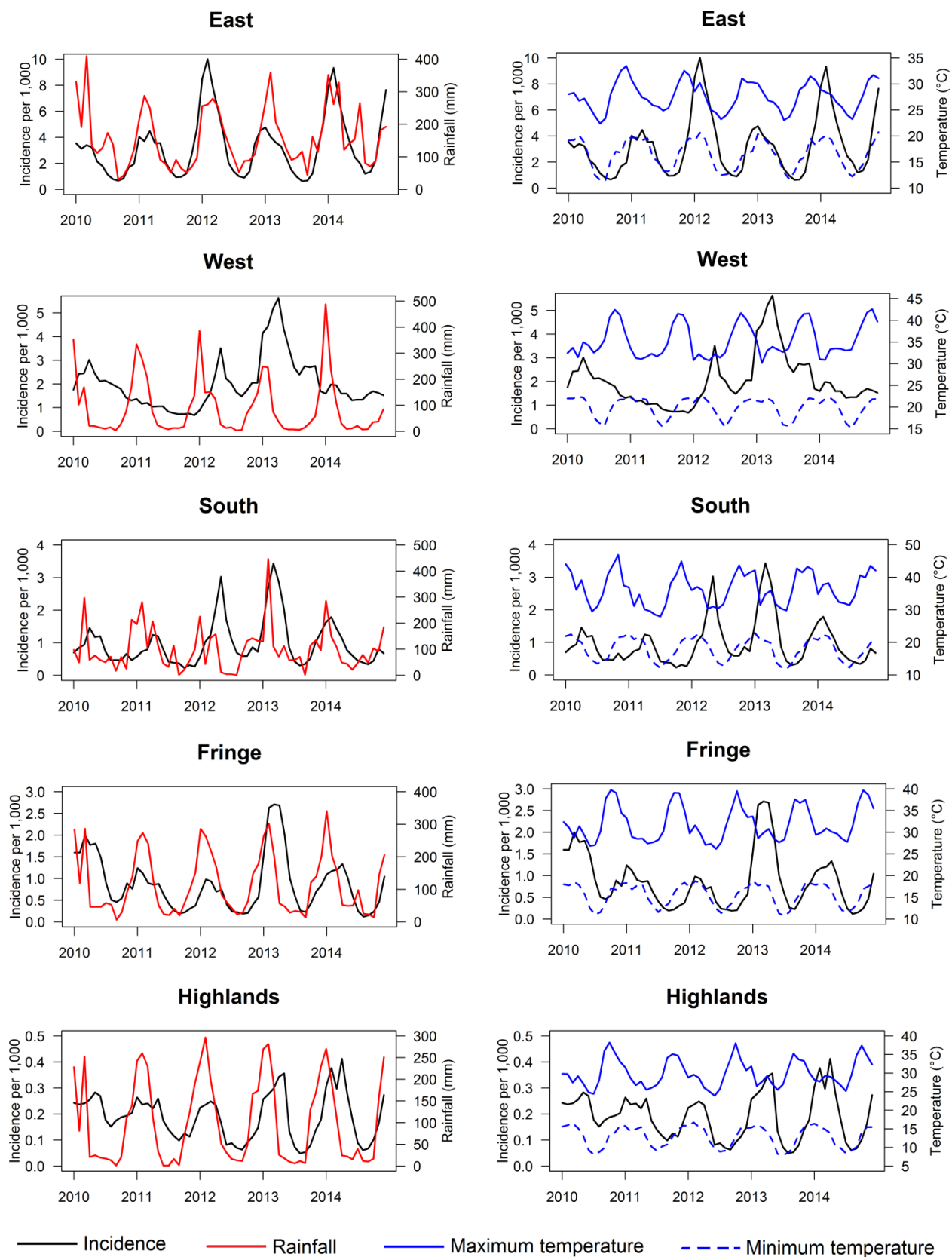

Supplement: Supplementary file 6 — Additional file 6. Malaria incidence, rainfall and temperature between 2010 and 2014. [file 12936_2018_2206_MOESM6_ESM.pdf]
