# Supplementary material for: Spatial and temporal dynamics of malaria in Madagascar
Source: Malar J. 2018 Feb 1;17:58. doi: 10.1186/s12936-018-2206-8 (PMC5796477; doi:10.1186/s12936-018-2206-8)
Supplement: Supplementary file 3 — Additional file 3. Number of districts per malaria intensity within stratification. The West has the most number of district within stratification and the South the lowest. The number of district with high incidence increased across the year mainly in 2013 within stratification. [file 12936_2018_2206_MOESM3_ESM.pdf]

Additional file 3: Number of districts per malaria intensity within stratification. The West has the most number of district within stratification and the South the lowest. The number of district with high incidence increased across the year mainly in 2013 within stratification.

Stratum	Year	n					
		**Low**	**Lower**	**Lowest**	**High**	**Higher**	**Highest**
East (n= 29)	2010	0	5	10	12	2	0
	2011	2	6	8	9	4	0
	2012	4	4	7	11	2	1
	2013	2	6	8	10	3	0
	2014	6	4	6	9	4	0
West (n= 39)	2010	4	4	14	11	6	0
	2011	4	5	9	17	4	0
	2012	4	5	12	12	6	0
	2013	4	1	15	13	6	0
	2014	5	3	8	17	6	0
South (n=7)	2010	1	3	1	0	2	0
	2011	0	2	2	3	0	0
	2012	0	3	2	1	1	0
	2013	1	0	4	1	1	0
	2014	1	2	2	1	1	0
Fringe (n=16)	2010	2	5	4	3	1	1
	2011	2	4	2	3	5	0
	2012	2	4	3	4	2	1
	2013	1	2	4	5	4	0
	2014	2	3	1	7	2	1
Highlands (n=20)	2010	7	8	2	2	1	0
	2011	5	6	6	2	1	0
	2012	1	4	8	6	1	0
	2013	1	2	9	7	1	0
	2014	4	5	6	2	3	0
Total		65	96	153	168	69	4

n: number of district
