# Supplementary material for: Spatial and temporal dynamics of malaria in Madagascar
Source: Malar J. 2018 Feb 1;17:58. doi: 10.1186/s12936-018-2206-8 (PMC5796477; doi:10.1186/s12936-018-2206-8)
Supplement: Supplementary file 4 — Additional file 4. Malaria clustering using the retrospective spatial analysis. [file 12936_2018_2206_MOESM4_ESM.pdf]

Additional file 4: Malaria clustering using the retrospective spatial analysis

Stratum	Year	Type	N	RR	LLR	p-value	District name
East	2010	1	8	1.65	3,170.10	0.00	
		2	1	2.29	2,860.91	0.00	Taolagnaro
		3	1	3.04	1,238.59	0.00	AntanambaoManampotsy
		4	1	1.27	94.33	0.00	Marolambo
		5	1	1.17	48.27	0.00	Brickaville
		6	1	1.09	15.55	0.00	Toamasina II
	2011	1	5	2.56	12,764.99	0.00	
		2	4	1.82	4,609.89	0.00	
		3	1	2.24	2,229.13	0.00	Ifanadiana
		4	1	1.71	791.74	0.00	Marolambo
	2012	1	15	2.73	28,479.19	0.00	
		2	1	1.55	1,193.71	0.00	Toamasina II
	2013	1	13	2.82	21,633.81	0.00	
		2	1	1.12	47.90	0.00	Toamasina II
	2014	1	15	3.12	42,857.07	0.00	
		2	1	1.18	213.13	0.00	Taolagnaro
West	2010	1	15	2.81	13,515.79	0.00	
		2	1	1.37	49.28	0.00	Benenitra
		3	1	1.18	39.62	0.00	Miandrivazo
	2011	1	20	2.02	2,969.37	0.00	
		2	1	1.99	238.82	0.00	Ankazoabo
		3	2	1.20	27.85	0.00	
		4	1	1.17	27.08	0.00	Ambilobe
	2012	1	17	2.67	11,607.36	0.00	
		2	1	1.50	246.49	0.00	Morondava
		3	1	1.30	31.45	0.00	Benenitra
		4	1	1.10	11.14	0.00	Morombe
	2013	1	23	2.16	12,506.40	0.00	
		2	2	1.42	750.55	0.00	
		3	1	1.30	112.21	0.00	Mahajanga II
	2014	1	19	2.27	6,437.067	0.00	
		2	1	2.26	1,264.24	0.00	Analalava
		3	1	1.29	79.43	0.00	Antsohihy
		4	1	1.38	76.99	0.00	Mahajanga II
		5	1	1.30	76.47	0.00	Tsaratanana
		6	1	1.15	29.50	0.00	Port-Berge (Boroziny-Vaovao)
South	2010	1	2	7.48	5,620.87	0.00	
	2011	1	3	3.83	1,982.88	0.00	
	2012	1	1	4.53	4,901.60	0.00	AmboasaryAtsimo
		2	1	1.95	628.96	0.00	Bekily
	2013	1	1	3.64	3,623.23	0.00	Bekily
	2014	1	3	5.09	4456.20	0.00	

Continued

Additional file 4: continued

Stratum	Year	Type	N	RR	LLR	p-value	District name
Fringe	2010	1	1	6.64	6,185.41	0.00	Anosibe-An'Ala
		2	1	3.77	5,725.62	0.00	Tsiroanomandidy
		3	1	1.35	159.18	0.00	Betafo
	2011	1	1	4.79	1,729.24	0.00	Anosibe-An'Ala
		2	1	2.25	866.01	0.00	Tsiroanomandidy
		3	2	1.56	181.04	0.00	
		4	1	1.48	143.49	0.00	Moramanga
		5	1	1.44	115.77	0.00	Betafo
		6	1	1.52	48.02	0.00	Andilamena
	2012	1	2	3.88	2,585.47	0.00	
		2	1	2.77	655.42	0.00	Mandoto
		3	1	2.68	229.61	0.00	Iakora
		4	1	1.46	31.84		Andilamena
		5	1	1.28	10.40	0.00	Ivohibe
	2013	1	1	2.78	1,636.62	0.00	Mandoto
		2	1	2.97	1,260.86	0.00	Anosibe-An'ala
		3	3	1.63	527.46	0.00	
		4	1	2.05	349.29	0.00	Andilamena
		5	2	1.42	234.22	0.00	
		6	1	1.12	23.11	0.00	Tsiroanomandidy
	2014	1	9	2.46	18.68	0.00	
		2	1	1.56	115.76	0.00	Ankazobe
		3	1	1.51	48.77	0.00	Andilamena
		4	1	1.25	43.87	0.00	Moramanga
Highlands	2010	1	1	9.37	16,337.85	0.00	Antananarivo Renivohitra
		2	1	1.33	37.71	0.00	Soavinandriana
	2011	1	1	5.48	6,751.65	0.00	Antananarivo Renivohitra
		2	1	1.59	78.73	0.00	Soavinandriana
	2012	1	1	2.00	574.11	0.00	Antananarivo Renivohitra
		2	1	2.59	369.97	0.00	Ambositra
		3	3	1.49	114.88	0.00	
		4	1	1.93	108.91	0.00	Fianarantsoa I
	2013	1	1	5.38	1,927.12	0.00	Ambositra
		2	1	1.75	92.77	0.00	Ambohimahasoa
		3	1	1.46	42.41	0.00	Miarinarivo
		4	1	1.41	36.34	0.00	Ambatolampy
		5	1	1.31	29.34	0.00	Antananarivo Avaradrano
		6	2	1.18	18.82	0.00	
	2014	1	5	5.45	5,428.68	0.00	
		2	1	2.81	450.58	0.00	Soavinandriana

Type: 1: the primary cluster; 2, 3, 4, 5, 6, 7: secondary clusters
N: number of district was detected by retrospective space-time analysis
RR: Relative risk; LLR: Log likelihood ratio
