# Supplementary material for: Spatial and temporal dynamics of malaria in Madagascar
Source: Malar J. 2018 Feb 1;17:58. doi: 10.1186/s12936-018-2206-8 (PMC5796477; doi:10.1186/s12936-018-2206-8)
Supplement: Supplementary file 5 — Additional file 5. Malaria clustering using the retrospective space-time analysis. [file 12936_2018_2206_MOESM5_ESM.pdf]

Additional file 5: Malaria clustering using the retrospective space-time analysis

Year	Stratum	Time frame	Type	N	RR	LLR	p-value	District name
2010	East	January-May	1	15	2.40	9,423.64	0.00	
	East	January-June	2	1	3.61	4,858.05	0.00	Taolagnaro
	East	December	3	1	2.95	671.75	0.00	Vangaindrano
	West	February-July	1	14	2.91	12,197.14	0.00	
	West	April-July	2	1	2.50	212.21	0.00	Benenitra
	South	February-July	1	2	6.85	4,736.00	0.00	
	Fringe	January-June	1	1	10.87	7,131.47	0.00	Anosibe-An'Ala
	Fringe	February-July	2	1	4.34	4,514.72	0.00	Tsiroanomandidy
	Highlands	January-June	1	1	6.62	9,795.57	0.00	Antananarivo Renivohitra
	Highlands	April-June	2	1	1.21	106.10	0.00	Soavinandriana
2011	East	January-May	1	6	3.34	14,459.87	0.00	
	East	November-December	2	4	3.00	4,557.27	0.00	
	West	January-June	1	18	2.15	3,088.58	0.00	
	South	January-June	1	3	4.44	2,443.35	0.00	
	South	April-May	2	1	2.30	71.23	0.00	Tsihombe
	Fringe	January-May	1	5	3.99	3,521.30	0.00	
	Highlands	February-July	1	1	4.93	4,537.51	0.00	Antananarivo Renivohitra
	Highlands	January-May	2	1	2.81	242.51	0.00	Soavinandriana
	Highlands	January-April	3	1	1.96	89.81	0.00	Ambositra
	Highlands	April-May	4	1	1.71	19.70	0.00	Fianarantsoa I
2012	East	January-April	1	15	4.05	53,727.07	0.00	
	West	March-August	1	17	2.74	10,706.59	0.00	
	South	February-July	1	1	6.24	5,598.45	0.00	AmboasaryAtsimo
	South	April-May	2	1	5.74	1,791.54	0.00	Bekily
	South	May	3	3	1.38	41.94	0.00	
	Fringe	January-May	1	2	7.13	4,027.62	0.00	
	Fringe	March-May	2	3	1.77	166.02	0.00	
	Highlands	January-May	1	1	3.04	989.31	0.00	Antananarivo Renivohitra
	Highlands	January-April	2	12	2.01	501.03	0.00	
2013	East	November-December	1	12	3.35	16,855.47	0.00	
	East	January-April	2	1	1.38	146.13	0.00	Antalaha
	East	March-April	3	1	1.28	30.44	0.00	MananaraAvaratra
	West	January-June	1	23	2.35	14,889.50	0.00	
	West	February-July	2	2	1.94	1,664.14	0.00	

Continued

Additional file 5: continued

Year	Stratum	Time frame	Type	N	RR	LLR	p-value	District name
2013	South	January-May	1	1	6.76	5,823.35	0.00	Bekily
	South	March-April	2	2	2.22	739.53	0.00	
	South	March-May	3	3	1.67	354.26	0.00	
	Fringe	January-May	1	9	3.23	5,410.33	0.00	
	Fringe	February-May	2	5	2.39	2,167.18	0.00	
	Highlands	January-May	1	6	4.53	2,563.18	0.00	
	Highlands	May	2	1	9.70	780.69	0.00	Antananarivo Avaradrano
	Highlands	April-May	3	2	5.32	612.28	0.00	
	Highlands	January-May	4	3	1.73	121.85	0.00	
	Highlands	November	5	1	2.99	120.79	0.00	Antananarivo Atsimondrano
2014	East	January-April	1	14	3.14	40,937.33	0.00	
	West	January-June	1	7	2.38	3,026.48	0.00	
	West	July-December	2	3	2.68	1,161.34	0.00	
	West	February-July	3	2	1.92	696.10	0.00	
	West	January-March	4	6	1.45	491.43	0.00	
	South	January-May	1	3	4.73	4,125.68	0.00	
	South	January	2	1	2.06	41.77	0.00	Tsihombe
	Fringe	January-May	1	9	3.54	3274.93	0.00	
	Fringe	January-May	2	2	2.28	396.49	0.00	
	Highlands	January-May	1	5	8.06	6556.91	0.00	
	Highlands	February-June	2	1	5.44	827.57	0.00	Soavinandriana
	Highlands	April-May	3	3	1.57	51.40	0.00	
	Highlands	December	4	1	2.03	25.50	0.00	Antanifotsy
	Highlands	January	5	1	1.77	8.47	0.044	Andramasina

Type: 1: the primary cluster; 2, 3, 4, 5: the secondary clusters
N: number of district was detected by retrospective space-time analysis
RR: relative risk; LLR: log likelihood ratio
